# Morphology of male World Cup and elite speed climbers

**DOI:** 10.3389/fspor.2025.1679627

**Published:** 2025-10-30

**Authors:** Paweł Draga, Paulina Baran, John Henry Maskell, Paulina Trybek, Dominik Pandurevic, Alexander Sutor

**Affiliations:** ^1^ Department of Biomedical Informatics and Mechatronics, Private University for Health Sciences, Medical Informatics and Technology (UMIT), Hall in Tirol, Austria; ^2^ WIR gemeinnutzige GmbH, Hall in Tirol, Austria; ^3^ JM Coastal, Wrea Green, United Kingdom; ^4^ Institute of Physics, University of Silesia in Katowice, Chorzów, Poland

**Keywords:** speed climbing, competitive climbing, body composition, kinanthropometry, somatotype

## Abstract

**Aims:**

To compare somatic characteristics and somatotypes of elite World Cup and national level speed climbers relative to general adult population norms, and to identify anthropometric traits that differentiate performance levels.

**Materials and methods:**

Eighteen male speed climbers participated in the study, including 10 international level and 8 national level athletes. Anthropometric data were collected according to the ISAK protocol, and somatotype was determined using the Heath-Carter method. Statistical analyses included the Shapiro–Wilk test to assess normality, the Student’s *t*-test or Mann–Whitney *U* test to compare groups. Principal component analysis (PCA) to reduce dimensionality and identify body composition characteristics differentiating athletes by performance level, and Spearman’s rank correlation coefficient to examine relationships between variables.

**Results:**

International climbers showed significantly lower body fat (6.46%±1.22% vs. 9.40%±1.46%), and higher lean body mass (93.5%±1.22% vs. 90.6%±1.46%). They exhibited wider biacromial breadth (42.98±1.98 cm vs. 41.03±1.18 cm), humeral breadth (7.67±0.40 cm vs. 6.93±0.50 cm), and femoral breadth (9.49±0.44 cm vs. 8.99±0.42 cm). Both groups presented an ectomorphic-mesomorphic somatotype, with international athletes displaying a significantly higher mesomorphic component (6.08±0.81 vs. 4.63±0.61).

**Conclusions:**

International climbers differ from national-level athletes by having lower fat mass, greater lean body mass, and greater skeletal breadth, including biacromial, humeral, and femoral widths. Both groups show substantial morphological differences compared to the general adult population. Differences in the breadth of the humerus and femur, as well as in biacromial width, may reflect specific adaptations to the load patterns typical of speed climbing. Athletes at the international level showed a more homogeneous somatic profile, indicating morphological optimization at the highest levels of performance. Traits considered important in other climbing disciplines were not found to be relevant in speed climbing. The results presented require verification in larger and more diverse groups of speed climbers. Nevertheless, with appropriate caution, they may serve as an initial reference point for talent identification and morphological optimization in speed climbing.

## 1 Introduction

A new format was implemented for the Olympic Games starting in 2024, combining lead climbing and bouldering, while speed climbing remained an individual competition (1). However, although speed climbing is a separate, individual climbing discipline, there seems to be less research on it compared to other competitive climbing formats. This limited number of studies may be due to the lower number of athletes practicing speed climbing, both professionally and recreationally. Nevertheless, because of its spectacular character and the possibility of setting official world records on a fully standardized 15 m route, speed climbing has attracted scientific interest (2). Its dynamic and spectacular nature only adds to its attractiveness. A comprehensive analysis of the influence of individual somatic and motor factors on the sport level of elite speed climbers was performed by Krawczyk et al. (3). Among the variables influencing climbers’ results, he identified explosive strength of the lower limbs and maximal anaerobic power as significant factors. Research into speed climbing has mostly been directed towards clarifying the effect of upper limb power (4) and lower limb power (5) on sports performance. The ability to use modern technology and marker-free movement tracking also made it possible to analyze movement technique and its relationship to athletes’ performance (6).

Researchers often point out body morphology as an important factor influencing athletic performance (7). It is commonly described by size and segment lengths (height, limb lengths, body mass), proportions (anthropometric indices), and tissue composition (fat and lean mass). In the context of the influence of body morphology on athletic outcomes, not only the athlete’s composition, size, and mass are important, but also the relative proportions of these components, which can vary depending on the athlete’s level of proficiency (8). Beyond the aforementioned morphological characteristics, researchers define the somatotype, which is described as a method for the quantitative assessment of body shape and composition (9), and is expressed as three numbers that can be plotted on a somatogram (10). The Heath-Carter method (11), one of the most widely used approaches to determining somatotype, accounts for tissue composition, body size, and proportions in three components: endomorphy (fatness), mesomorphy (muscularity), and ectomorphy (slenderness) (12).

The impact of somatic build on performance in speed climbing was examined in a study by Krawczyk et al. (13), who demonstrated that speed climbers show higher values of body mass, height, lean body mass, Rohrer index, and BMI compared to athletes specialized in bouldering and lead. Levernier et al. (4), in a comparative analysis of the three main climbing disciplines, confirmed these tendencies, reporting that speed climbers are characterized by greater body mass, body fat percentage, and biacromial breadth. However, this comparative analysis provided limited information on the broader spectrum of morphological traits, which may differ between groups of athletes due to the different nature of effort (14). A literature review indicates a limited availability of extensive data on the body composition of speed climbers, particularly regarding somatotype classification based on the Heath-Carter method (15). Furthermore, the limited number of comprehensive studies and the variation in anthropometric methods make it difficult to compare data between different levels of sporting proficiency and disciplines within sport climbing.

Therefore, the aim of this study was to compare somatic components and somatotype profiles of elite international and national level speed climbers using standardized anthropometric protocols (16), and to identify body composition features that are relevant to speed climbing.

## 2 Materials and methods

The study included 10 international climbers ranked within the TOP 16 of the IFSC, representing the following countries: Ukraine (3), Russia (2), and one athlete each from Poland, Iran, Italy, France, and the Czech Republic. An additional group of 8 athletes consisted of Polish National Cup finalists. Participants were recruited by email, provided detailed study information and gave voluntary consent. Inclusion criteria were male athletes aged 18 to 35 years with at least five years of speed climbing experience. National athletes had to compete in at least three national events annually and hold national team status within the last two years. International athletes met the same criteria and also had to participate in at least three World Cup or World Championship events annually over the past two years, with at least one placement in the Top 16. All data were anonymized. Anthropometric assessments took place during competitions at different times of the day, taking into account the pre competition context.

**Figure 1:**
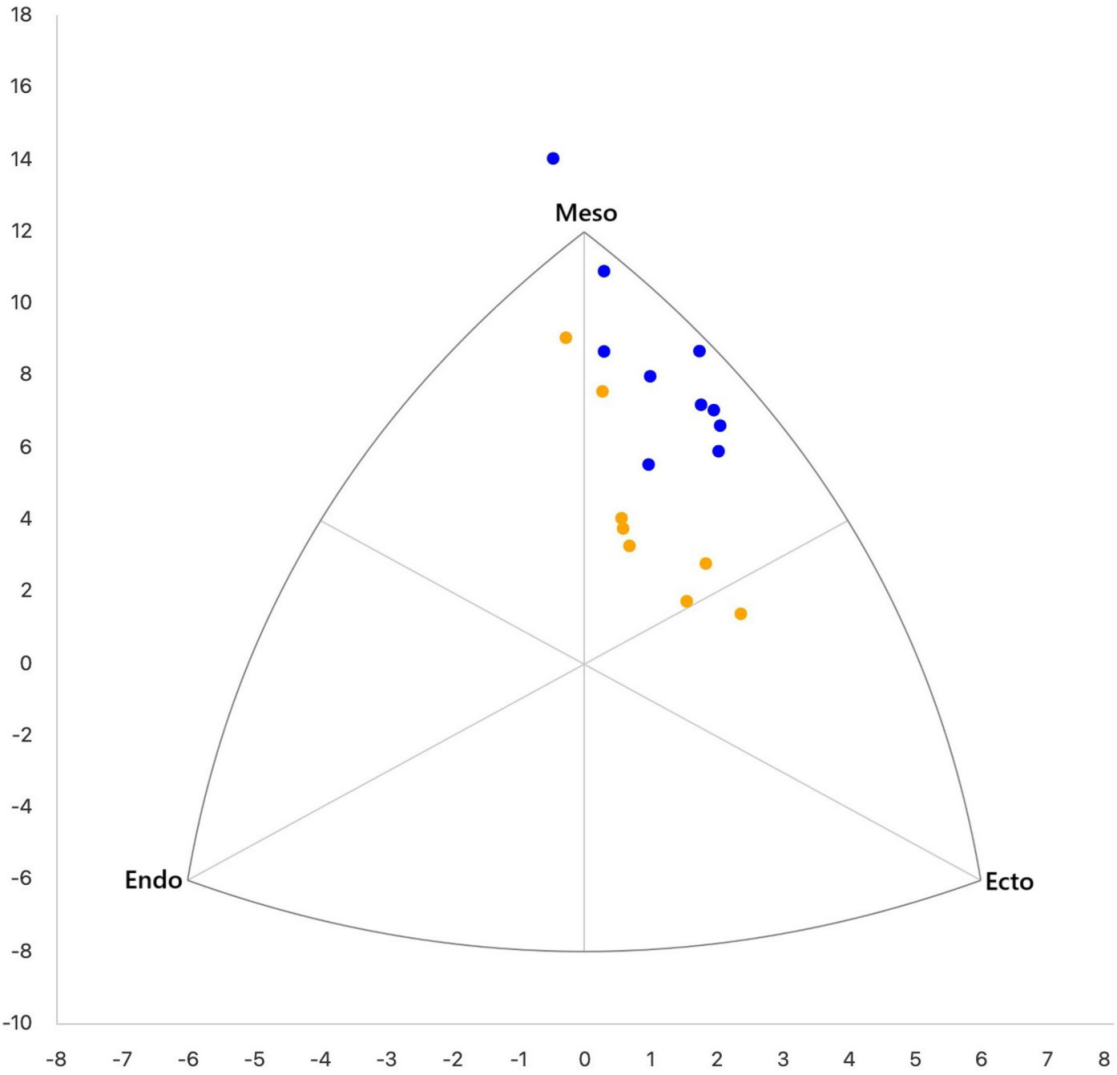
Somatotype profiles of international (blue) and national (yellow) speed climbers.

Measurements were performed by the same trained researcher, who completed a one year internship in anthropometry and physical profiling at the National Research Institute (Institute of Sport). The internship included practical training in standardized measurement protocols aligned with ISAK guidelines (16), and the full list of assessed variables is presented in Table 1. Somatotype classification was performed using the Heath Carter method (15), as shown in Figure 1. Data were collected using the following instruments: an anthropometer (SiberHegner, Switzerland; precision: ±0.1 cm) for body height; skinfold calipers (Harpenden, Baty International, UK; ±0.2 mm) for skinfold thickness; a circumference measuring tape (Seca 201, Seca GmbH & Co. KG, Germany; ±0.1 cm) for body circumferences; a small bone caliper (SiberHegner, Switzerland; ±0.1 cm) for wrist and bicondylar diameters; and a digital scale (Tanita TBF-583, Japan; ±0.1 kg) for body weight. Fat percentage was estimated using the Keys and Brožek method (17), with body density calculated according to the Durnin and Womersley equation (18). Data were collected by a researcher trained by the National Research Institute (Institute of Sport) in anthropometry and physical profiling, including practical training in standardized measurement protocols (16). Measurement repeatability and intra evaluator reliability were controlled using the technical coefficient of variation (TCV%), calculated from three non consecutive measurements at each site. Based on established standards (19, 20), thresholds were set at 1% for girths and 5% for skinfolds. Measurements that met these limits were used in the analysis.

**Table 1:** Anthropometric variables measured (abbreviations): X=Σ TS, SbS, SpS × 170.18 ÷ BH; AG, CG, corrected girths (arm, calf); HWR, height-to-weight ratio; a, B, v, tr, sst, sy, $da_{3}$, anatomical landmarks.

Category	Variable	Formula/measurement
Lengths & indices	Body height [BH] (cm)	Vertex to floor
	Arm length [AL](cm)	Acromiale to dactylion
	Leg length [LL](cm)	Trochanterion to floor
	Arm span [AS](cm)	Dactylion to dactylion
	Torso length [TL](cm)	Suprasternale to symphysion
	Arm index [ArI]	$\frac{a-da_{3}}{\left(B-v\right)}\cdot 100$ (27)
	Ape index [ApI]	$\frac{da_{3}-da_{3}}{\left(B-v\right)}$ (28)
	Leg index [LI]	$\frac{\left(B-tr\right)}{\left(B-v\right)}\cdot 100$ (27)
	Torso index [TI]	$\frac{sst-sy}{\left(B-v\right)}\cdot 100$ (27)
	Intermembral index [II]	$\frac{a-da_{3}}{\left(B-sy\right)}\cdot 100$ (27)
Breadths	Shoulder [SB](cm)	Acromiale to acromiale
	Pelvic [PB] (cm)	Iliocristale to iliocristale
	Humerus [HB](cm)	Epicondylion laterale to epicondylion mediale
	Femur [FB](cm)	Epicondylion laterale to epicondylion mediale
Girths	Forearm [FG] (cm)	Midpoint between wrist and elbow
	Arm tensed [ATG](cm)	Maximal circumference during contraction
	Arm relaxed [ARG] (cm)	Midpoint of relaxed arm
	Waist [WG] (cm)	Narrowest part of torso
	Thigh [TG] (cm)	Midpoint between inguinal crease and patella
	Calf [CG] (cm)	Maximal calf circumference
	Neck [NG] (cm)	Below laryngeal prominence
	Chest Inh. [CIG] (cm)	Maximal chest expansion
	Chest exhalatio [CEG] (cm)	At end of normal expiration
Skinfolds & body composition	∑ of 7 Skinfolds (mm)	TS, BS, SbS, AS, CS, PS, TS
	Triceps [TS] (mm)	Vertical fold, midline posterior upper arm
	Biceps [BS](mm)	Vertical fold, midline anterior upper arm
	Pectoral [PS] (mm)	Diagonal fold, mid-chest
	Subscapular [SbS](mm)	Diagonal fold, below inferior angle of scapula
	S.iliac [SiS](mm)	Diagonal fold, above iliac crest
	Abdominal [AS](mm)	Vertical fold, 2 cm from umbilicus
	Calf [CS](mm)	Vertical fold, medial calf
	S.spinale [SpS](mm)	Diagonal fold, above anterior superior iliac spine
	Thigh [TS](mm)	Vertical fold, midline anterior thigh
	Body weight [BW](kg)	
	Body mass index [BMI]	$\frac{\text{BW}}{(B-v{)}^{2}}$ (29)
	Density [D](g/cm${}^{3}$)	$1.1631-0.0632\cdot \log_{10}(B+T+Sb+SiS)$ (18)
	Fat mass [FM] (%)	$100\times \left(\frac{4.201}{D}-3.813\right)$ (17)
	Fat mass [FM] (kg)	From fat mass percentage
	Lean body mass [LBM](%)	$100\times \left(1-\frac{\text{FM\textbackslash{}\%}}{100}\right)$
	Lean body mass [LBM](kg)	Body weight–fat mass
	Rohrer’s index [RI]	$\frac{\text{BW}}{(B-v{)}^{3}}$ (30)
Somatotype (15)	Endomorphy [Endo]	$-0.7182+0.1451X-0.00068X^{2}+0.0000014X^{3}$
	Mesomorphy [Meso]	0.858⋅HB+0.601⋅FB+0.188⋅AG+
		0.161⋅CG−0.131⋅BH+4.5
	Ectomorphy [Ecto]	0.732⋅HWR−28.58ifHWR≥40.75

The comparative analysis used combined reference data from four studies in the general adult population (21–24), due to the lack of a single comprehensive data set. For normally distributed variables (p>0.05), an independent *t* test was used; for non normal distributions (p<0.05), the Mann Whitney *U* test was applied. Principal Component. Principal Component Analysis (PCA) was applied to reduce a large set of correlated variables into a smaller number of uncorrelated components, explaining most of the variability in the data. PCA was performed on variables differentiating national and international speed climbers to identify morphological patterns and determine the morphological profile of elite speed climbers (25). Spearman correlations were computed per group for selected variables (see Supplementary Table S1), replacing individual skinfolds with sum of skinfolds, FM%, and FM (kg) to reduce redundancy. The study complied with local ethics guidelines and the Declaration of Helsinki (26).

## 3 Results

**Figure 2:**
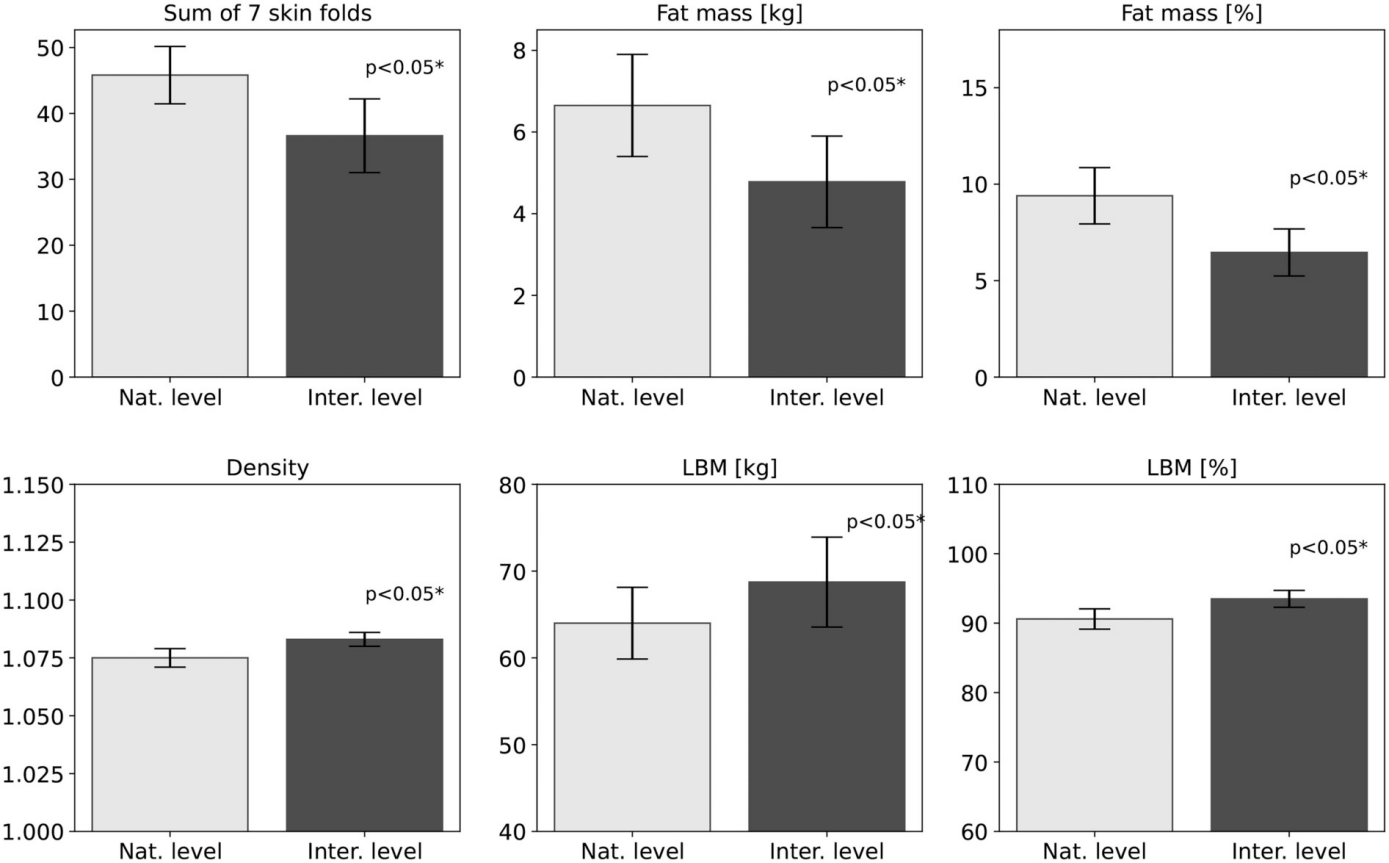
Differences in body fat, skinfolds, lean body mass, and density between international and national speed climbers. Bar charts are shown with standard deviations of the mean.

**Figure 3:**
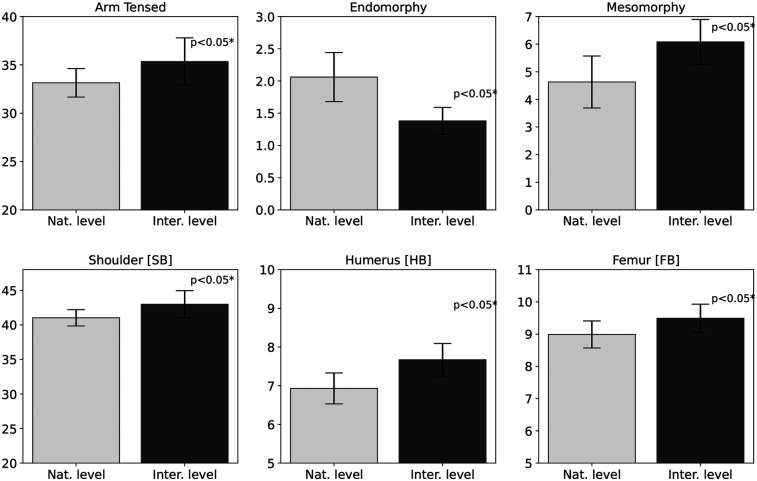
Differences in somatotype components, tensed arm circumference, and skeletal breadths between international and national speed climbers. Bar charts are shown with standard deviations of the mean.

Statistically significant differences between international-level speed climbers and national-level athletes were identified in 16 variables (see Table 2). The largest disparities concerned fat-related parameters. The suprailiac skinfold thickness was 50.2% (p=0.023) lower in the international group, and the fat mass% was reduced by 45.5% (p=0.001). Similar patterns were observed in other skinfolds, indicating lower fat levels among international athletes. Significant differences were also observed in somatotype components, with the international group showing 49.3% lower endomorphy (p=0.001) and 23.8% higher mesomorphy (p=0.003) compared to the national group. Body density was 0.74% (p=0.007) higher in international athletes, representing the smallest yet statistically significant difference. Structural variables also differed: humerus 9.65% (p=0.003), femur 5.27% (p=0.030), and biacromial breadth 4.53% (p=0.030) were all greater in this group. LBM was 6.82% (p=0.037) higher in the international group, which was also reflected in arm tensed girth 6.22% (p=0.004). The most relevant variables are presented in bar charts (Figures 2, 3), grouped by component type, with standard deviations included to illustrate variability in the group.

**Table 2:** Descriptive statistics for somatic and demographic variables in international (Int.) and national (Nat.) athletes.

Variable	Mean ± SD	Mean ± SD	Min–Max	Min–Max	% change	*p*-value	TCV%
	Nat. (*n* = 8)	Int. (*n* = 10)	Nat.	Int.			
Body height [BH] (cm)	178.34 ± 6.12	177.98 ± 8.21	168.5–186.9	167.5–188	0.20	0.451	–
Arm length [AL] (cm)	80.16 ± 2.78	79.33 ± 3.74	75.4–83.9	74.0–84.6	1.04	0.447	–
Leg length [LL] (cm)	84.36 ± 4.05	85.57 ± 1.97	77.5–90.0	76.3–95.7	1.43	0.267	–
Arm span [AS] (cm)	184.44 ± 6.23	185.2 ± 2.14	175.7–193.5	177.0–195.0	0.41	0.809	–
Torso length [TL] (cm)	52.03 ± 0.90	51.94 ± 1.10	49.0–55.2	46.3–56.8	0.17	0.955	–
Arm index [ArI]	45.27 ± 0.72	44.60 ± 0.49	43.64–50.06	42.50–46.69	1.50	0.439	–
Leg index [LI]	52.27 ± 0.47	51.94 ± 0.41	50.01–53.70	49.67–53.73	0.64	0.604	–
Torso index [TI]	29.34 ± 0.32	29.20 ± 0.43	27.28–31.21	28.04–30.68	0.48	0.798	–
Intermembral index [II]	86.10 ± 1.38	85.88 ± 0.95	82.28–95.17	80.38–90.29	0.26	0.898	–
APE index [ApI]	1.03 ± 0.01	1.04 ± 0.01	1.00–1.07	0.98–1.07	0.97	0.590	–
Biacromial [SB] (cm)	41.03 ± 1.18	42.98 ± 1.98	39.7–43.2	39.0–46.0	4.53	**0.030**	–
Pelvic [PB] (cm)	28.56 ± 1.92	27.49 ± 1.22	25.3–31.0	25.9–30.0	3.89	0.160	–
Humerus [HB] (cm)	6.93 ± 0.40	7.67 ± 0.42	6.5–7.8	7.0–8.0	9.65	**0.003**	–
Femur [FB] (cm)	8.99 ± 0.42	9.49 ± 0.44	8.4–9.6	9.0–10.1	5.27	**0.030**	–
Forearm [FG] (cm)	28.83 ± 1.20	29.65 ± 1.01	27.0–31.0	28.0–31.0	2.76	0.138	0.73
Arm tensed [ATG] (cm)	33.14 ± 1.47	35.34 ± 2.45	31.4–35.6	31.0–39.5	6.22	**0.040**	0.91
Arm relaxed [ARG] (cm)	29.83 ± 1.46	31.91 ± 2.49	28.0–31.5	28.0–35.5	6.52	0.053	0.74
Waist [WG] (cm)	75.99 ± 2.63	78.29 ± 3.30	73.0–79.7	72.5–82.5	2.93	0.128	0.95
Thigh [TG] (cm)	54.81 ± 2.67	54.68 ± 2.31	52.0–60.5	50.0–58.5	0.24	0.911	0.90
Calf [CG] (cm)	36.74 ± 1.60	37.07 ± 1.68	35.0–39.8	34.9–41.0	0.90	0.675	0.70
Neck [NG] (cm)	36.89 ± 1.00	37.20± 1.78	35.5–38.5	34.5–40.0	0.83	0.665	0.86
Chest Inh. [CIG] (cm)	94.78 ± 3.71	94.65 ± 3.89	90.3–102.0	87.5–98.0	0.14	0.963	0.92
Chest Exh. [CEG] (cm)	87.59 ± 4.04	87.25 ± 3.74	82.3–94.5	82.0–92.5	0.39	0.856	0.81
Sum of 7 skinfolds (mm)	45.81 ± 4.35	36.61 ± 5.59	38.4–51.0	28.3–45.5	25.1	**0.002**	–
Triceps [TS] (mm)	5.63 ± 0.90	4.54 ± 1.00	4.6–7.6	3.5–6.8	24.0	**0.033**	4.06
Biceps [BS] (mm)	3.51 ± 0.10	2.78 ± 0.65	2.6–5.4	2.0–3.5	26.3	0.155	3.14
Pectoral [PS] (mm)	5.95 ± 0.96	5.45 ± 0.99	4.4–7.6	4.0–7.0	9.17	0.295	4.14
S. scapular [SbS] (mm)	8.33 ± 1.60	6.18 ± 1.40	6.4–10.2	3.0–8.1	34.8	**0.007**	4.06
S. iliac [SiS] (mm)	7.30 ± 2.90	4.86 ± 1.10	4.6–13.8	3.0–6.5	50.2	**0.023**	5.14
Abdominal [AS] (mm)	7.16 ± 1.60	6.59 ± 1.60	4.2–9.1	4.0–8.8	8.65	0.458	5.20
Calf [CS] (mm)	3.67 ± 0.16	3.63 ± 0.30	3.50–3.98	3.0–4.1	6.88	0.702	3.57
S. spinale [SpS] (mm)	5.46 ± 3.80	3.37 ± 0.90	3.0–14.7	2.5–5.0	38.3	**0.033**	4.81
Thigh [TS] (mm)	7.94 ± 2.16	6.21 ± 1.32	4.0–10.2	4.5–9.0	27.9	0.052	4.86
Body Weight [BW] (kg)	70.7 ± 4.66	73.51 ± 6.00	66.9–80.0	60.5–78.5	4.68	0.288	–
BMI (kg/$m^{2}$)	22.24 ± 1.31	23.21 ± 1.45	20.8–24.4	21.2–26.3	4.17	0.160	–
Density [D] (g/${\mathrm{cm}}^{3}$)	1.08 ± 0.004	1.08 ± 0.003	1.07–1.08	1.08–1.09	0.74	**0.007**	–
FM (%)	9.40 ± 1.46	6.46 ± 1.22	7.17–11.51	5.05–8.58	45.5	**0.001**	–
FM (kg)	6.65 ± 1.25	4.78 ± 1.12	5.18–9.20	3.06–6.65	39.1	0.001	–
LBM (%)	90.6 ± 1.46	93.5 ± 1.22	88.5–92.8	91.4–94.5	3.2	**0.004**	–
LBM (kg)	64.0 ± 4.13	68.73 ± 5.18	60.38–70.79	57.44–73.62	6.82	**0.037**	–
Roher’s Index [RI]	1.25 ± 0.10	1.31 ± 0.13	1.2–1.4	1.2–1.6	4.6	0.054	–
Endomorphy [Endo]	2.06 ± 0.38	1.38 ± 0.21	1.53–2.54	1.58–1.81	49.3	**0.001**	–
Mesomorphy [Meso]	4.63 ± 0.94	6.08 ± 0.81	3.4–6.4	5.1–7.8	23.8	**0.003**	–
Ektomorphy [Ekto]	3.00 ± 0.85	2.54 ± 0.95	1.76–3.88	0.7–3.63	18.1	0.292	–
Age (years)	22.27 ± 2.80	25.30 ± 4.11	19–29	20–33	15.4	0.304	–

Significant differences are shown in bold and highlighted in gray.

### 3.1 Principal component analysis (PCA)

**Figure 4:**
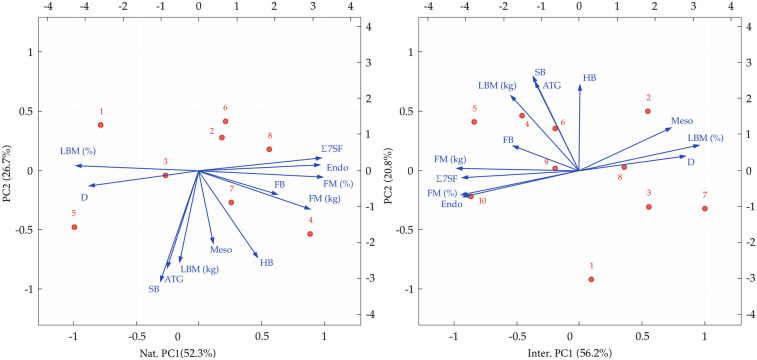
PCA results for national (left) and international (right) speed climbers, showing somatotype components and selected anthropometric variables.

The first two principal components (PC1 and PC2) explained 77%–79% of the total variance in both groups of athletes. The biplot shows (see Figure 4) the structure of the variables and the location of the athletes in the principal component space. The first principal component explained a similar percentage of variance in the international (56.2%) and national (52.3%) groups, contrasting features related to fatness and leanness (percentage body fat, fat mass in kg, endomorphy, and ∑7 skinfolds loaded negatively; body density, LBM, and mesomorphy loaded positively, with slightly stronger positive associations in the national group). The second principal component explained 20.8% of the variance in the international group and showed positive associations with biacromial breadths, body density, arm tensed girths, and LBM (kg), reflecting variation in muscularity and upper body dimensions. In the national group, PC2 explained 26.7% of the variance and showed negative associations with the same variables. The opposite directions of variable loads for PC2 on the biplot suggest differences in morphology between speed climber groups.

## 4 Discussion

Anthropometric studies of national and international speed climbers have shown clear differences between groups of athletes and compared to the general adult population. A comparative analysis showed that the most pronounced differences between climber groups were in body composition. International-level athletes had significantly lower body fat levels, both in terms of percentage and total skinfold thickness. Significant differences in parameters such as tensed arm girth and body density were observed, which were higher in international climbers. These athletes also had larger skeletal dimensions, particularly in the humerus, femur, and biacromial breadths. The international speed climbers presented somatotypical differences compared to the national speed climbers, characterized by less endomorphism and greater mesomorphism. The group of international speed climbers was also characterized by greater homogeneity in morphology, which may indicate preferred body type patterns in this discipline. Anthropometric parameters such as body height, body weight, and ape index, considered important by researchers in bouldering (8) and lead climbing (31), did not differentiate speed climbers. This finding may indicate different morphological requirements for achieving success at the highest level in speed climbing.

### 4.1 Body fat assessment

Significant differences between speed climbers were observed between groups as well as compared to the general adult population. The average body fat content, both in relative values (6.45%±1.21%vs.9.39%±1.45%) and absolute values (4.78±1.12kgvs.6.65±1.25kg), was lower for international speed climbers (Table 2). Principal component analysis showed that variables related to body fat (fat mass, skin fold thickness, endomorphism) clustered and were opposite to lean mass and body density in the international group, indicating a distinct low fat somatotype (see Figure 4). This relationship was not observed in national speed climbers, where PCAs were more spread and did not follow a distinct somatic build pattern as in the international group of speed climbers. The values obtained are similar to those reported by Krawczyk et al. (3); in their study, the average body fat content was 7.62% in elite male speed climbers. However, the studies cited did not show any significant correlation between body fat levels and the athletic performance of speed climbers. These results contradict the findings of studies conducted in athletes in lead and bouldering (8, 32–34), in which body fat was identified as a factor that significantly influences athletic performance. Referring to the general adult population, where Kalka et al. (21) reported an average body fat content of (18.4%±2.9%), both groups of climbers included in the study show significantly lower values. The results indicate that low body fat, while characteristic of the morphological profile of both international and national speed climbers, does not alone ensure athletic success (35) and may have negative consequences for health (36). Monitoring and modification of this component of body composition should therefore be conducted under the supervision of sports medicine and nutrition professionals within a holistic approach.

### 4.2 Lean body mass

The present study shows that LBM was significantly different between climbers competing at the international and national levels, with relative values of (93.5%±1.22%) and (90.06%±1.46%), and absolute values of (68.7%±5.18kg) and (64.04±4.13kg). Greater lean body mass in international climbers may support higher generation of strength and power, which is important in speed climbing. Although LBM differentiated performance levels, analysis of body girths revealed only one significant difference in tensed arm girths, where international level climbers had larger values ((35.34±2.45cm)) compared to national level climbers ((33.14±1.47cm)). No significant differences in the girth of other limbs or the torso were found between the groups. The higher lean body mass observed in international climbers corresponds with their increased body density, likely due to larger circumferences of selected body segments. The results obtained are higher than those observed in other climbing disciplines. In lead climbing, competitors achieved an average LBM value of (47.2kg) (37), and in bouldering, (57.8kg) (38). The lack of data makes it impossible to compare the percentage values of LBM with other studies involving speed climbers. This parameter can be compared with data obtained by Draga et al. (8), but in relation to elite boulder climbers. In this comparison, speed climbers are characterized by both a higher percentage and absolute lean body mass. The values obtained in both groups of speed climbers are also higher than those recorded in the general adult population, such as (62.59%) LBM reported by Żarów et al. (22). These results consistently confirm the thesis that sports requiring high physical effort promote the development of greater muscle mass compared to endurance sports and physical inactivity (39–41). The data collected indicate that body density values were higher than those reported by Ozimek et al. (42) in bouldering athletes ($1.06\pm 0.008\;{g/\mathrm{cm}}^{3}$) and by España-Romero et al. (43) in lead climbers ($1.04\pm 0.06\;g/cm^{3}$). No data are available for direct comparison with speed climbers. In relation to other sports disciplines, the body density of speed climbers is similar to that observed in sprinters, hurdlers, decathletes, and jumpers (44). From a population perspective, the values recorded in speed climbers are significantly higher than those observed in the general adult population, where body density is approximately ($1.05\;{g/\mathrm{cm}}^{3}$) (23, 45). In comparison with other climbing disciplines, the girth measurements, especially of the thighs and calves, were noticeably higher in speed climbers (46). This observation is confirmed by a study conducted by Krawczyk et al. (47), which showed that speed climbers significantly develop muscle groups specifically involved in speed climbing. The researchers called this pattern regional muscle hypertrophy, a term that describes targeted muscle growth in areas subjected to high repetitive mechanical stress, such as the thighs and calves in speed climbing. These observations are supported by studies conducted by Carrasco et al. (48), who found greater forearm LBM in elite lead climbers compared to intermediate climbers, assessed using DXA scans. These results are consistent with previous findings (46–48). These observations indicate the need to develop lean body mass in muscle regions involved in speed climbing, while noting that excessive growth may increase body mass and impair performance. Although relative strength and power were not directly measured, previous studies indicate their importance for speed climbing performance (5).

### 4.3 Skeletal breadth

The breadth of the biacromial, humerus, and femur differentiated international climbers from national climbers (see Table 2). Significant differences in humeral breadth (7.67±0.42 cm vs. 6.93±0.40 cm) and femoral breadth (9.49±0.44 cm vs. 8.99±0.42 cm) favored international climbers (Table 2). Principal component analysis showed that biacromial breadth had a significant impact on the second principal component (PC2), explaining 20.8% and 26.7% of the variance in the international and national groups, respectively, reflecting differences in upper body stature and muscularity. These observations are consistent with previous studies. Reyepko (49) reported that speed climbers had greater biacromial breadth (37.14±2.22 cm) compared to all-around climbers (35.1±1.62 cm), potentially linked to higher lean body mass and bone or muscle density (50, 51). Humeral values aligned closely with those reported by Mora-Fernández et al. (52), while femoral values were slightly lower. Relative to normative data (24), humeral breadth in both groups exceeded the general population average (6.8±0.6 cm), whereas femoral breadth was slightly lower (9.9±0.7 cm), suggesting sport-specific bone adaptations resulting from remodeling processes, particularly in the upper limbs. Similar adaptations were reported by Kemmler et al. (53), who observed increased bone mineral density in climbers exposed to high mechanical stress. Considering the observed bone adaptations and the dynamic nature of speed climbing, implementing targeted mechanical stimuli, such as strength and plyometric exercises, is important for supporting bone adaptation and potentially enhancing athletic performance (54).

### 4.4 Somatotype

Statistically significant differences were observed between two groups of speed climbers in two somatotype components, with lower endomorphy (1.38±0.21vs.2.06±0.38) and higher mesomorphy (6.08±0.81vs.4.63±0.94) in the international group (see Table 2 and Figure 1). Based on the values of the individual somatotype components, both groups can be classified as ectomorphic-mesomorphic, with a dominant mesomorphic profile that is reflected in their high lean body mass and low fat mass. A comparison of speed climbers’ somatotypes with other studies is not possible due to the lack of generally available such data. In the context of other climbing disciplines, however, somatotype has been the subject of research, providing conflicting findings regarding its impact on sport performance (42, 55, 56). Fernández-Mora et al. (52) reported a negative effect of endomorphy on performance in lead climbing, as well as lower mesomorphy values among elite lead climbers (5.61±0.58) compared to the results presented here. The higher mesomorphy values observed in speed climbers may be explained by the more strength and power oriented nature of this climbing discipline, which may favor the development of this somatotype component (57). According to Stanković et al. (56), the ectomorphic component does not significantly affect success in sport climbing, which partially aligns with the findings of this study, as ectomorphy did not differentiate between the analyzed groups. In comparison to the general adult population (24), endomorphy values in both climbing groups were considerably lower (3.6±0.17), mesomorphy was higher only in the international group (4.9±1.2), while ectomorphy (2.2±1.0) was lower than in both climbing groups. The results indicate significant somatotype-based differentiation of speed climbers depending on their level of performance, although further verification is required in larger study samples.

## 5 Conclusions

An analysis of the speed climbers’ morphology demonstrated significant differences between international and national athletes and the general adult population, indicating body somatic patterns characteristic of elite athletes in this discipline. It was found that international level climbers are characterized by a more homogeneous somatic structure, including lower body fat content, higher lean body mass, higher body density, and larger skeletal dimensions, as well as more pronounced mesomorphy and reduced ectomorphy. The study also noted significant differences in the breadth of the humerus and femur, and in biacromial width, which may result from adaptation of the skeletal system to specific load patterns found in speed climbing. The results obtained can serve as a reference point for talent identification in this discipline and for optimizing body composition. They may also provide practical guidance for coaches in supporting the long-term athlete development of athletes with respect to the desired somatic profile in speed climbing. However, it should be noted that such a detailed anthropological analysis of speed climbing has not been performed before, and it was not possible to compare the results with broader data sets, which limits the generalizability of the findings. Therefore, more research is necessary on larger groups of speed climbers with different levels of sports performance to verify the results presented, especially given that the characteristics considered important in other climbing disciplines did not appear to be relevant for performance in speed climbing.

## 6 Strengths and limitations

The study has several limitations that must be considered. The small sample of participants, including only male athletes, makes it difficult to apply the results to a larger population of climbers. The results do not allow us to clearly state whether the physical profile that characterizes the best speed climbers is the result of adaptation to training or genetic predisposition. Data on training regimens and nutrition were also not reviewed during the investigation, and their influence on the findings is unknown. Logistical constraints due to the field nature of the measurements prevented the use of more advanced measurement techniques. The participants were mainly from Europe, but they had different ethnic origins and socio-cultural backgrounds, the impact of which was not taken into account in the study. Despite its limitations, the study has notable strengths. It provides rare and valuable data on the somatic profiles of the best international speed climbers. The standardized measurement protocol used, with a comprehensive range of anthropometric variables, contributes to understanding the basic somatic factors in speed climbing. Methodological compatibility allows for the comparison of speed climbers with bouldering athletes (8).

## Data Availability

The raw data supporting the conclusions of this article cannot be made publicly available due to participant privacy and data protection regulations. Anonymized aggregated data are available from the authors upon reasonable request.
