# Supplementary material for: Morphology of male World Cup and elite speed climbers
Source: Front Sports Act Living. 2025 Oct 30;7:1679627. doi: 10.3389/fspor.2025.1679627 (PMC12611891; doi:10.3389/fspor.2025.1679627)
Supplement: Supplementary file 1 [file Datasheet1.pdf]

SUPPLEMENTAL DATA

Table 3. Significant ($p < 0.05$) correlations between anthropometric and body composition variables in national and international speed climbers.

Group	Variable A	Variable B	r	p-value
**National**				
	Biacromial	Arm Tensed	0.805	0.016
	Biacromial	LBM (kg)	0.827	0.011
	Humerus	FM (kg)	0.774	0.024
	Arm Tensed	Mesomorphy	0.816	0.013
	$\sum$ of 7 skinfolds	Density	-0.885	0.004
	$\sum$ of 7 skinfolds	FM (%)	0.969	< 0.001
	$\sum$ of 7 skinfolds	FM (kg)	0.839	0.009
	$\sum$ of 7 skinfolds	LBM (%)	-0.969	< 0.001
	$\sum$ of 7 skinfolds	Endomorphy	0.938	0.001
	Density	FM (%)	-0.850	0.007
	Density	LBM (%)	0.850	0.007
	Density	Endomorphy	-0.826	0.012
	FM (%)	FM (kg)	0.924	0.001
	FM (%)	LBM (%)	-1.000	< 0.001
	FM (%)	Endomorphy	0.968	< 0.001
	FM (kg)	Endomorphy	0.863	0.006
	LBM (%)	Endomorphy	-0.968	< 0.001
**International**				
	Biacromial	Femur	0.695	0.026
	Arm Tensed	LBM (kg)	0.753	0.012
	$\sum$ of 7 skinfolds	Density	-0.764	0.010
	$\sum$ of 7 skinfolds	FM (%)	0.903	< 0.001
	$\sum$ of 7 skinfolds	FM (kg)	0.940	< 0.001
	$\sum$ of 7 skinfolds	LBM (%)	-0.903	< 0.001
	$\sum$ of 7 skinfolds	Endomorphy	0.900	< 0.001
	$\sum$ of 7 skinfolds	Mesomorphy	-0.681	0.030
	Density	FM (%)	-0.854	0.002
	Density	FM (kg)	-0.832	0.003
	Density	LBM (%)	0.854	0.002
	Density	Endomorphy	-0.797	0.006
	Density	Mesomorphy	0.661	0.037
	FM (%)	FM (kg)	0.961	< 0.001
	FM (%)	LBM (%)	-1.000	< 0.001
	FM (%)	Endomorphy	0.990	< 0.001
	FM (%)	Mesomorphy	-0.721	0.019
	FM (kg)	Endomorphy	0.948	< 0.001
	FM (kg)	Mesomorphy	-0.679	0.031
	LBM (%)	Endomorphy	-0.986	< 0.001
	LBM (%)	Mesomorphy	0.721	0.019
	Endomorphy	Mesomorphy	-0.697	0.024
